# Asperger Traits as a Conditionally Adaptive Neurocognitive Phenotype: An Evolutionary Narrative Review

**DOI:** 10.7759/cureus.112205

**Published:** 2026-07-07

**Authors:** Alexander Bertuccioli, Annalisa Belli, Chiara Maria Palazzi, Francesco Di Pierro, Ilaria Cavecchia, Mariarosaria Matera

**Affiliations:** 1 Biomolecular Sciences Department, University of Urbino Carlo Bo, Urbino, ITA; 2 Coordination Department, Microbiota International Clinical Society, Turin, ITA; 3 Administration Department, Microbiota International Clinical Society, Turin, ITA; 4 Management Department, Microbiota International Clinical Society, Turin, ITA; 5 Scientific &amp; Research Department, Velleja Research, Milan, ITA; 6 Medicine and Technological Innovation Department, University of Insubria, Varese, ITA; 7 Microbiomic Department, Koelliker Hospital, Turin, ITA; 8 Pediatric Emergencies Department, Misericordia Hospital, Grosseto, ITA

**Keywords:** autism spectrum disorder, biological evolution, cognition, epigenetics, genetic epistasis, neurodiversity

## Abstract

Traits historically associated with Asperger syndrome, currently included within autism spectrum disorders (ASD), include high systemizing ability, sustained attention to detail, reduced reliance on social cues, and strong cognitive perseverance. Although these characteristics are often interpreted as impairments within contemporary social environments, an increasing body of literature suggests that they may also reflect variants of human cognitive diversity with potential evolutionary relevance. This narrative review explores whether the cognitive profile commonly associated with Asperger traits can be interpreted within an evolutionary framework, integrating perspectives from evolutionary genetics, cognitive neuroscience, and developmental biology. A targeted selection of theoretical and experimental contributions was examined through full-text analysis, including models related to epistatic genetic architecture, neurocognitive network organization (including the default mode network), evolutionary behavioral strategies such as the “solitary forager” hypothesis, the extreme male brain theory, genomic imprinting models of cognition, and recent approaches investigating epistatic interactions in autism-related genetic pathways. Findings were synthesized through conceptual triangulation within a narrative review framework. Across these models, several convergent themes emerge. Traits associated with the Asperger phenotype, such as enhanced systemizing, cognitive autonomy, persistence, and technical imagination, may represent extreme expressions of cognitive strategies that could have been advantageous in certain ancestral ecological contexts. In small and variable environments, such traits may have supported technological innovation, environmental exploration, and resilience under conditions requiring independent problem-solving. Genetic evidence suggests that autism-related phenotypes arise from complex polygenic and epistatic networks rather than single-gene alterations, supporting the possibility that these traits reflect stable variants within human neurocognitive diversity. Emerging research also indicates a potential modulatory role of intergenerational epigenetic mechanisms in shaping neurodevelopmental trajectories, although these interpretations remain theoretical and should be considered with caution. Overall, the Asperger phenotype may be interpreted not solely as a neurodevelopmental deficit but as a context-dependent cognitive configuration whose functional impact varies according to environmental demands. In modern societies characterized by high social communication load, rapid relational dynamics, and sensory overstimulation, these traits may contribute to an evolutionary mismatch. However, the interaction between polygenic architecture, epistatic regulation, and potential epigenetic modulation suggests that the persistence of such cognitive phenotypes may reflect broader mechanisms maintaining neurocognitive diversity within the human population.

## Introduction and background

Asperger syndrome is no longer recognized as an independent diagnostic category in the Diagnostic and Statistical Manual of Mental Disorders, Fifth Edition (DSM-5) and the DSM-5, Text Revision (DSM-5-TR) and is currently subsumed under autism spectrum disorder (ASD) [1]. In this review, the term “Asperger” is therefore used exclusively in a historical and descriptive sense. The unit of analysis is not Asperger syndrome as a separate nosographic entity but a cluster of cognitive and behavioral traits historically associated with individuals previously diagnosed with Asperger syndrome or with autistic profiles without intellectual disability or major language impairment [2]. These traits may include, in some individuals, strong systemizing, sustained attention to detail, cognitive perseverance, pattern-focused reasoning, technical interests, sensory sensitivity, and lower reliance on implicit social cues [2]. However, these features are heterogeneous, non-universal, and may coexist with functional impairment, sensory distress, psychiatric comorbidity, communication difficulties, executive dysfunction, and variable support needs [3,4]. Accordingly, the expression “Asperger-associated traits” is used throughout the manuscript as a pragmatic historical descriptor, not as a biologically discrete phenotype or as a diagnostic category separate from ASD. The unit of analysis is therefore a non-universal cognitive trait cluster, rather than a diagnosis, a homogeneous subgroup, or a discrete biological phenotype.

The aim of this narrative review is to integrate selected theoretical and empirical contributions in order to explore whether Asperger-associated traits may be interpreted as context-dependent expressions of neurocognitive variability while explicitly avoiding causal claims about adaptive selection or evolutionary maintenance [5-10]. The review does not seek to demonstrate that these traits are universally adaptive or that they were directly selected for in ancestral environments [5,7]. Rather, it examines whether models from evolutionary genetics, cognitive neuroscience, developmental biology, and autism research may contribute to a hypothesis-generating framework in which the functional impact of these traits depends on environmental demands [5-12]. Within this perspective, characteristics such as systemizing, persistence, pattern-focused reasoning, technical imagination, and lower reliance on implicit social cues may be considered neither inherently advantageous nor inherently pathological but context-sensitive features whose outcomes vary across ecological, social, developmental, and support-related conditions [2,3,5,10].

## Review

Methods

The present work was designed as a narrative review aimed at exploring whether selected cognitive traits historically associated with Asperger syndrome, currently subsumed under ASD, may be interpreted within a context-dependent evolutionary and neurobiological framework. The review was not intended to provide a systematic or meta-analytic assessment of the literature nor to estimate effect sizes, prevalence, or causal relationships. Rather, it sought to integrate selected theoretical and empirical contributions relevant to the relationship between autism-related cognitive traits, neurocognitive variability, evolutionary hypotheses, genetic architecture, and emerging intergenerational biological mechanisms.

A targeted literature search was conducted in PubMed and Scopus up to April 2026. Search terms included combinations of the following: “autism spectrum disorder,” “Asperger syndrome,” “autistic traits,” “neurodiversity,” “systemizing,” “evolutionary psychiatry,” “evolutionary autism,” “epistasis,” “polygenic architecture,” “default mode network,” “mirror neuron system,” “imprinted brain hypothesis,” “extreme male brain theory,” “solitary forager,” “paternal epigenetics,” “sperm DNA methylation,” and “intergenerational epigenetics.” Additional references were identified through citation chaining from relevant reviews and theoretical papers.

Because the purpose of the article was conceptual integration rather than systematic evidence grading, papers were selected according to theoretical relevance, disciplinary complementarity, and their contribution to one or more predefined conceptual domains: evolutionary genetics, neurocognitive network organization, evolutionary behavioral strategies, systemizing-based cognitive models, genomic imprinting hypotheses, molecular genetics of ASD, epidemiology, strengths-based autism research, and intergenerational epigenetics. Priority was given to peer-reviewed articles in English, foundational theoretical models, recent empirical studies, and reviews directly relevant to the interpretative framework. No formal risk-of-bias assessment or quantitative evidence grading was performed. Therefore, the selection of studies may primarily reflect conceptual relevance to the proposed framework and may not capture the full range of competing interpretations in autism research.

Studies were excluded from the central synthesis when they did not address autism-related cognition, evolutionary or neurobiological interpretation, genetic or epigenetic mechanisms, or functional outcomes relevant to the aims of the review. The review also avoided treating broad ASD risk literature as direct evidence for Asperger-associated cognitive profiles unless this distinction was explicitly discussed.

The synthesis was conducted through conceptual triangulation across levels of analysis. Specifically, evidence was compared across genetic, epigenetic, neurobiological, cognitive, evolutionary, and contextual domains. Each contribution was classified according to its evidentiary status: theoretical model, narrative or systematic review, molecular or genetic study, neuroimaging study, epidemiological study, or strengths-based autism-outcome research. This classification was used to distinguish hypothesis-generating interpretations from correlational findings and from evidence directly testing mechanisms. Accordingly, the present review should be read as an integrative and exploratory narrative framework rather than as a systematic demonstration of adaptive function. The key conceptual domains, representative references, level of evidence, role in the review, and main limitations are summarized in Table 1.

**Table 1:** Key conceptual domains, representative studies, main findings, level/type of evidence, and limitations included in the narrative synthesis. DMN: default mode network; ASD: autism spectrum disorders

Conceptual domain	Key references	Level/type of evidence	Role in the review	Main limitation
Neurodiversity and strengths-based framing	[2,13-15]	Review/empirical outcomes	Supports a non-exclusively deficit-based interpretation	Does not eliminate impairment or support needs
Evolutionary approaches to autism	[5,7]	Theoretical/evolutionary models	Provides hypothesis-generating evolutionary interpretations	Does not directly test ancestral fitness
Epistasis and genetic architecture	[6,16,17]	Theoretical/molecular genetics	Supports polygenic and epistatic complexity	Does not demonstrate adaptive maintenance
DMN and self-processing	[9,10,18,19]	Review/neuroimaging	Candidate neurobiological correlates	Not specific to Asperger-associated traits
Systemizing and technical cognition	[20,21]	Cognitive theory/empirical psychology	Frames systemizing and rule-based cognition	Controversial and incomplete as a general autism model
Imprinted brain hypothesis	[8]	Theoretical model	Explores parent-of-origin hypotheses	Not causally established for ASD profiles
Imagination and autistic cognition	[22,23]	Cognitive theory/review	Distinguishes technical from social imagination	Indirect evidence for context-dependent functional relevance
Intergenerational epigenetics	[11,12,24-27]	Review/associative molecular and epidemiological studies	Possible modulatory mechanisms	Associative, preliminary, not Asperger-specific
Epidemiology and modern contexts	[28-33]	Epidemiology/systematic reviews	Contextualizes prevalence and modern niches	Does not establish evolutionary mechanisms

Results 

Epistasis Hypothesis and Neurocognitive Variability

Ijichi and colleagues proposed that traits commonly described as autistic may be interpreted as the extreme tail of the quantitative distribution of human cognitive traits and that such characteristics may be influenced by stabilizing epistasis [6]. Within this hypothesis, autism-related cognitive traits are not interpreted as the result of a single developmental anomaly but as the possible outcome of complex interactions among multiple genetic loci and regulatory factors [6,16,17]. In the present review, this model is considered a hypothesis-generating framework for understanding how certain cognitive configurations historically associated with Asperger syndrome may persist within broader human neurocognitive variability.

According to this perspective, the evolution of highly cooperative human groups may not have eliminated more independent or idiosyncratic cognitive styles. Under selected ecological or social conditions, traits such as systemizing, perseverance, attention to environmental regularities, and lower reliance on social consensus may have offered potential functional relevance [5,7,20,21]. For example, such traits could hypothetically have supported pattern detection, technical problem-solving, environmental exploration, or the maintenance of cognitive diversity within small groups [5,7,20,21]. However, these interpretations remain theoretical and should not be understood as direct evidence that Asperger-associated traits were adaptively selected or that they consistently increased individual or group fitness. This interpretation is broadly compatible with evolutionary approaches suggesting that diversity in cognitive strategies may contribute to group flexibility under changing environmental conditions [5]. Nevertheless, the specific role of epistasis in maintaining Asperger-associated cognitive profiles remains inferential. Molecular genetic studies have shown that ASD is highly polygenic and involves multiple interacting genes and regulatory networks [16,17]. Such findings support the complexity of autism-related genetic architecture, but they do not demonstrate that specific cognitive traits historically associated with Asperger syndrome were maintained by selection. Therefore, epistasis is discussed here as a possible contributor to neurocognitive variability, rather than as an established mechanism explaining the evolutionary persistence of a discrete Asperger-associated trait profile. Accordingly, epistasis should be considered a biological framework for heterogeneity, not evidence of adaptive preservation.

Taken together, these observations suggest that Asperger-associated traits may be situated within a broader spectrum of human neurocognitive variability [2,3,5,6,16,17]. Their functional significance, however, should be interpreted as context-dependent, probabilistic, and heterogeneous, rather than as evidence of a universally adaptive profile.

Paralimbic Network, Default Mode Network (DMN), and Self-Processing

The review by Molnar-Szakacs and Uddin examined the relationship between the DMN and the mirror neuron system, two neural systems involved in self-referential processing, social cognition, and the mental simulation of others’ states [9,18]. The DMN includes medial prefrontal, posterior cingulate/precuneus, and temporo-parietal regions, and is particularly active during internally oriented cognitive processes such as introspection, autobiographical reflection, and mentalizing [9,18]. Neuroimaging studies have reported altered functional connectivity and dynamics within the DMN in ASD, particularly in regions involved in self-referential and social-cognitive processing [10,19].

These findings may be relevant to the present review because they suggest that some autistic profiles are associated with differences in the balance between internally oriented cognition and social-cognitive processing [9,10,18,19]. Some studies have reported reduced integration between the DMN and systems involved in social simulation, which may contribute to differences in self-other representation, mentalizing, and social responsiveness [9,18]. From an evolutionary perspective, one could hypothesize that relatively greater autonomy of internally oriented processing may have been functionally relevant in situations requiring sustained attention, independent decision-making, or tolerance of solitary activity [5,7,9,10]. However, this remains an interpretative hypothesis and should not be treated as a direct inference from neuroimaging data.

In contemporary societies, where implicit communication, rapid social adaptation, relational flexibility, and simultaneous processing of multiple socio-emotional cues are often required, such neurocognitive differences may contribute to functional difficulties. This possible mismatch should be understood as context-dependent: the same cognitive style may be more or less disabling depending on environmental demands, sensory load, social expectations, and available supports [2,3,7,20].

Importantly, altered DMN connectivity or dynamics should be interpreted here as a candidate neurobiological correlate reported in ASD research, not as a direct explanation of higher-order traits such as systemizing, technical imagination, perseverance, or independent problem-solving. The reviewed literature supports differences in self-referential and social-cognitive processing in some autistic individuals, but it does not establish a one-to-one mapping between DMN organization and the behavioral or cognitive traits discussed in this review [9,10,18,19]. Therefore, the relationship between DMN functioning, Asperger-associated traits, and context-dependent outcomes should be considered hypothesis-generating rather than mechanistically established.

The “Solitary Forager” Hypothesis as a Theoretical Evolutionary Model

Spikins and colleagues proposed an evolutionary interpretation according to which certain traits currently associated with the autism spectrum may correspond to alternative behavioral strategies relative to the highly social forms of cooperation that characterize homo sapiens [7]. Within this theoretical framework, human neurocognitive variability is not interpreted simply as deviation from a normative cognitive model, but as a possible source of differentiated behavioral strategies. The model suggests that, in some ancestral contexts, individuals with reduced dependence on group social dynamics, high attentional perseverance, and strong visuospatial or environmental monitoring abilities may have occupied complementary roles within small human groups [2,20].

In relatively small and mobile hunter-gatherer communities, such traits could hypothetically have supported more solitary exploratory activities, independent information gathering, attention to environmental detail, or technical problem-solving [5,7,20,21]. In this context, abilities such as sustained focus, systemizing tendencies, and sensitivity to environmental regularities may have had functional value under selected ecological conditions [20,21]. However, such claims remain indirect and theoretical. Current evidence does not allow one to determine whether these traits were directly selected for, whether they increased fitness, or whether they reflect by-products of broader neurodevelopmental variability. The “solitary forager” hypothesis is therefore best understood as a model of possible ancestral affordances rather than as a demonstrated evolutionary explanation. It helps conceptualize how cognitive styles less dependent on continuous social coordination might have been functional in some ecological niches, but it does not imply that such traits are universally advantageous or uniformly expressed across autistic individuals [3,5,7].

In contemporary society, many educational, occupational, and social contexts prioritize teamwork, implicit communication, rapid social flexibility, and continuous interpersonal coordination [3,4,7,13]. Under such conditions, more independent or less socially embedded cognitive strategies may become difficult to express adaptively and may instead contribute to distress, exclusion, or functional impairment [3,4,13-15]. This does not mean that the traits themselves are inherently maladaptive; rather, their impact depends on the fit between individual cognitive style, environmental demands, and available supports. Alternative explanations include non-adaptive by-products of neurodevelopmental variation, environmental mismatch, diagnostic ascertainment effects, and the selective visibility of strengths in structured modern contexts.

Extreme Male Brain Theory

According to the extreme male brain theory proposed by Baron-Cohen, autism can be interpreted as a cognitive profile characterized by a heightened tendency toward systemizing, defined as the drive to analyze, understand, and predict rule-governed systems [2,20]. Within this model, human cognition is described along two broad dimensions: empathizing, referring to the recognition and response to others’ mental and emotional states, and systemizing, referring to the identification of regularities, structures, and causal relationships within physical, logical, or abstract systems.

This framework is relevant to the present review because several traits historically associated with Asperger syndrome, including attention to detail, preference for rule-based systems, technical interests, and pattern-focused reasoning, overlap with the construct of systemizing [20,21]. From a context-dependent perspective, systemizing may support specific forms of analytical problem-solving and technical cognition [20-22]. In selected ecological or technological contexts, the ability to identify regularities, model causal relationships, and refine rule-governed procedures may have had functional value [5,7,20,21]. However, this does not demonstrate that systemizing was adaptively selected as part of an Asperger-associated profile.

The extreme male brain theory remains influential, but it should be considered a contested model rather than a definitive explanation of autism. Criticisms include the risk of overemphasizing sex-dimorphic cognition, underrepresenting heterogeneity within ASD, and insufficiently accounting for females, gender diversity, camouflaging, developmental variability, and variable support needs [3,4]. Moreover, systemizing alone cannot explain the full range of sensory, emotional, social, executive, and adaptive difficulties observed across the autism spectrum [3,4,20]. For this reason, the model should be used descriptively to discuss systemizing-related cognition, not inferentially to support adaptive or sex-essentialist conclusions.

In the present review, the extreme male brain theory is therefore used only as one possible framework for discussing systemizing and technical cognition. It should not be interpreted as a comprehensive model of autism, nor as evidence that Asperger-associated traits are uniformly adaptive or biologically fixed.

Imprinted Brain Hypothesis

The imprinted brain hypothesis proposes that some neurocognitive trajectories may be influenced by the balance between paternally and maternally derived genes subject to genomic imprinting, an epigenetic mechanism through which the expression of specific genes depends on the parent of origin [5,8]. Within this model, autism and psychotic disorders have been interpreted as opposite poles of social-cognitive variation. Autism has been hypothesized to reflect a relative bias toward paternal gene expression, whereas psychotic disorders have been hypothesized to reflect a relative bias toward maternal gene expression [8]. This model is relevant to the present review because it provides a theoretical framework for considering how parent-of-origin effects might contribute to neurocognitive variability. From this perspective, different balances in imprinted gene expression could hypothetically influence tendencies toward systemizing, social cognition, environmental control, or interpersonal sensitivity. However, this interpretation remains theoretical and should not be understood as a validated causal account of ASD or Asperger-associated traits.

The imprinted brain hypothesis is controversial and has important limitations. Complex neurodevelopmental phenotypes cannot be reduced to simple parent-of-origin effects, and current evidence does not establish a direct causal relationship between specific imprinting patterns and historically Asperger-like cognitive profiles [3,4,8]. In addition, the model risks oversimplifying the biological, developmental, and environmental heterogeneity of ASD [3,4,8].

Accordingly, the imprinted brain hypothesis is included here as a hypothesis-generating contribution to the broader discussion of neurocognitive diversity, not as evidence that Asperger-associated traits are determined by genomic imprinting or that they represent a discrete biological subtype [5,8]. It should therefore be interpreted as a conceptual comparator rather than as a mechanistic explanation.

Imagination, Social Cognition, and Autistic Trajectories

Crespi and colleagues proposed an interpretation of autistic cognition that distinguishes between different forms of imagination and mental simulation [22]. Within this framework, autism is not understood simply as a generalized deficit in imagination, but as a possible difference in the organization of imaginative processes. In some autistic individuals, cognitive activity may be more strongly oriented toward the systematic construction and manipulation of conceptual models than toward the spontaneous simulation of others’ emotional states or intentions [22,23]. This distinction is relevant to the present review because traits historically associated with Asperger syndrome may include technical interests, rule-based creativity, pattern-focused reasoning, and the construction of internally consistent conceptual systems. Such traits may support forms of technical or analytical imagination, particularly in contexts where structured reasoning, model-building, and systematic exploration are valued [20-23]. However, this should not be interpreted as a universal feature of autistic cognition, nor as evidence that technical imagination is generally superior to social imagination.

Within this model, the cognitive profile historically associated with Asperger syndrome can be described through several recurring but non-universal features: reduced spontaneous mental simulation of others’ mental states in some individuals, a marked tendency toward systematic combination of concepts, a form of creativity based on rules, patterns, and structural relationships, and strong internal or systematized coherence [2,20,22,23]. The latter expression is not used here in the classical sense of “central coherence” as described in autism cognitive theory, but rather to indicate a tendency to organize information according to internally consistent, rule-based, and pattern-focused structures. In selected technological, analytical, or problem-solving contexts, such cognitive tendencies may have functional relevance [13,15,20-22]. They may support the development of tools, strategies, symbolic systems, or technical solutions. Nevertheless, these interpretations remain context-dependent and cannot be generalized across the autism spectrum. In many social environments, a predominance of internally structured cognition may also be associated with difficulties in emotional interpretation, relational flexibility, implicit communication, and adaptive social participation [3,4,9,18,23].

Taken together, these observations suggest that autistic trajectories may involve not only difficulties in social processes, but also alternative modes of organizing imagination and abstract thought [2,20,22,23]. The functional meaning of these modes depends substantially on individual heterogeneity, environmental context, developmental stage, and available support.

Genetic Epistasis in Idiopathic Autism Pathways

The study by Mitra and colleagues employed reverse genetics approaches and molecular pathway analyses to investigate the genetic architecture of autism spectrum disorders [17]. Their findings support the view that autism-related traits cannot generally be attributed to isolated single-gene effects, but may emerge from complex networks of gene-gene interactions in which multiple genetic variants jointly contribute to phenotypic expression [16,17]. In this context, epistasis, understood as functional interaction among different genetic loci, may play a role in modulating neurodevelopmental trajectories associated with ASD [6,16,17].

This perspective is consistent with broader molecular genetic evidence indicating that ASD is highly polygenic and involves regulatory networks related to neuronal development, synaptic plasticity, transcriptional regulation, and other neurobiological pathways [16,17].

Such complexity supports the idea that autism-related phenotypes reflect multilocus and network-level processes rather than simple linear alterations of single genes [16,17].

For the purposes of the present review, these findings are relevant because they provide a biological basis for considering ASD-related traits as part of complex neurodevelopmental variability. However, the existence of polygenic and epistatic architecture does not demonstrate that specific Asperger-associated cognitive traits were adaptively selected or maintained because of functional advantages. The link between genetic complexity and evolutionary interpretation remains inferential.

Therefore, genetic epistasis should be discussed as a possible contributor to phenotypic heterogeneity and neurocognitive variability, not as proof of adaptive preservation of a discrete Asperger-associated trait profile [3,6,16,17]. Thus, genetic complexity should not be conflated with evolutionary adaptiveness. Future research integrating polygenic risk, gene-gene interactions, developmental trajectories, cognitive phenotyping, and environmental context will be necessary to clarify whether specific combinations of genetic variation are associated with distinct cognitive strengths, vulnerabilities, or context-dependent functional outcomes. 

Discussion

The theoretical and empirical contributions reviewed in this article suggest that traits historically associated with Asperger syndrome may be interpreted as part of broader human neurocognitive variability rather than exclusively through a deficit-oriented framework [2,3,6,17]. This interpretation is conceptual and does not imply that these traits are adaptive in themselves, adaptive in all individuals, or maintained by selection.

This framing, however, must remain cautious. The reviewed evidence does not demonstrate that Asperger-associated traits constitute an evolutionarily stable adaptive phenotype, nor that such traits were directly selected for in ancestral environments. Rather, the available evidence supports a hypothesis-generating framework in which genetic complexity, neurocognitive organization, cognitive style, developmental processes, and environmental context may interact to shape heterogeneous functional outcomes.

Within this framework, traits such as systemizing, persistence, pattern-focused reasoning, technical interests, sustained attention, and lower reliance on implicit social cues may have different functional meanings depending on context. In some structured or technically demanding environments, these traits may support effective problem-solving or specialized competence. In other contexts, particularly those characterized by high social ambiguity, sensory overload, rapid interpersonal demands, or limited accommodations, the same traits may contribute to distress, exclusion, or functional impairment [3]. Therefore, the central argument of this review is not that Asperger-associated traits are intrinsically adaptive, but that their functional significance is context-dependent, heterogeneous, and developmentally variable [2,3,7].

Controversies, Alternative Explanations, Heterogeneity, and Limits of Adaptive Interpretations

Several important cautions are necessary when interpreting Asperger-associated traits through an evolutionary framework. First, adaptationist explanations of complex neurodevelopmental phenotypes are intrinsically difficult to test. The fact that a trait may appear functionally useful under certain hypothetical ecological conditions does not demonstrate that it was directly selected for, nor that it increased individual or group fitness. Many of the models discussed in this review are theoretical, indirect, or based on extrapolation across different levels of analysis. Alternative explanations include developmental constraint, pleiotropy, non-adaptive variation, gene-environment interaction, ascertainment bias, and the possibility that some strengths become visible only in highly structured or supportive contexts. Therefore, the present framework should be considered hypothesis-generating rather than explanatory in a strict causal sense.

Second, some of the theories reviewed, including the extreme male brain theory and the imprinted brain hypothesis, remain influential but controversial. These models have contributed to the conceptual history of autism research, but they are not sufficient to account for the full heterogeneity of ASD. They may underrepresent sex and gender differences, camouflaging, developmental variability, environmental influences, psychiatric comorbidity, and the broad range of support needs observed across autistic individuals. Their inclusion in this review should therefore be understood as part of a comparative theoretical synthesis, not as an endorsement of a single explanatory model.

Third, a strengths-based or neurodiversity-informed interpretation must not romanticize autism-related suffering. Traits such as persistence, systemizing, attention to detail, and special interests may represent strengths in some individuals and contexts, but they can coexist with disabling sensory distress, anxiety, depression, social exclusion, communication difficulties, executive dysfunction, and substantial support needs. The functional meaning of these traits is therefore context-dependent, heterogeneous, and developmentally variable. A balanced framework must acknowledge both potential strengths and clinically relevant impairment.

Finally, caution is required to avoid biological essentialism. The concept of an “Asperger-associated cognitive profile” should not be interpreted as a fixed biological type or as a uniform pathway shared by all autistic individuals. Rather, it refers to a historically described cluster of traits that may be present to different degrees, may interact with environmental demands, and may produce different outcomes depending on support availability, contextual demands, developmental stage, and individual variability.

Intergenerational Epigenetic Influences: Preliminary and Associative Evidence

An additional emerging line of research concerns the possibility that parental environmental exposures may be associated with aspects of early neurodevelopment through germline epigenetic mechanisms [11,12]. Recent studies suggest that spermatozoa carry environmentally sensitive epigenetic information, including DNA methylation patterns, histone modifications, and small non-coding RNAs [11,12]. These mechanisms may contribute to early embryonic regulation and could represent one pathway through which paternal metabolic, nutritional, or environmental factors may be associated with influencing developmental outcomes [11,12].

However, this evidence must be interpreted with caution. Current studies linking paternal sperm methylation, environmental exposures, obesity, or other preconception factors to ASD-related outcomes are primarily associative [24-27]. They do not demonstrate that such mechanisms specifically generate Asperger-associated cognitive profiles, nor that they produce adaptive neurocognitive calibration [24-27]. Moreover, ASD risk-related epigenetic findings cannot be directly generalized to historically Asperger-associated traits such as systemizing, pattern-focused reasoning, technical imagination, or lower reliance on implicit social cues [20-27].

Therefore, in the present review, intergenerational epigenetic mechanisms are considered only as possible, non-specific modulators of neurodevelopmental variability within a broader polygenic and environmental framework [11,12,16,17,24-27]. They should not be interpreted as causal explanations of Asperger-associated traits. Future longitudinal studies integrating genomics, germline epigenomics, developmental neuroscience, and carefully characterized cognitive phenotypes will be needed to clarify whether such mechanisms contribute to specific neurodevelopmental trajectories [11,12,24-27].

Modern Microcontexts and Context-Dependent Functional Outcomes

The persistence and visibility of autistic traits in contemporary society can be considered through the lens of context-dependent functioning [2,3,7]. Modern environments often impose high demands for social flexibility, multitasking, implicit communication, sensory tolerance, and rapid adaptation to changing interpersonal contexts. For some individuals with Asperger-associated traits, these demands may increase the likelihood of mismatch, distress, fatigue, or functional impairment [3,4]. At the same time, certain modern microcontexts may provide conditions in which some traits can be expressed more constructively [2,13,14,15]. Structured digital environments, technical occupations, research settings, highly predictable routines, or communities organized around explicit rules may reduce reliance on implicit social cues and allow greater expression of systemizing, persistence, pattern detection, and specialized interests [13-15,20,21,28-30]. These contexts should not be interpreted as evidence of classical evolutionary adaptation [5,7]. Rather, they illustrate how environmental fit can modulate the functional impact of autistic traits [2,3,7].

This perspective is consistent with a dimensional and heterogeneity-based understanding of ASD [1,3,4]. The same trait may function as a strength, a vulnerability, or both, depending on the interaction among individual profile, developmental history, support needs, environmental structure, sensory load, social expectations, and available accommodations [2,3,13-15,34]. Therefore, the concept of evolutionary mismatch should be used cautiously: it may help frame some forms of difficulty in high-demand modern environments, but it does not provide a complete explanation of ASD nor a universal account of Asperger-associated profiles [3,5,7].

These modern microcontexts should not be interpreted as evidence that Asperger-associated traits are adaptive, but as examples of how environmental fit may modulate functional outcomes. Overall, the available literature supports a cautious model in which Asperger-associated traits are understood as context-dependent expressions of neurocognitive variability [2,3,5-7,13-15]. The model integrates theoretical, correlational, and preliminary evidence from multiple domains, but it does not establish a direct causal chain linking genetic architecture, neurobiological correlates, ancestral selection, and contemporary functioning [5-10,16-19,28-33]. 

Limitations

The main limitations of the present work derive from its narrative rather than systematic design. Although the review was expanded to improve transparency regarding search strategy, conceptual domains, and evidentiary classification, it does not provide a systematic or meta-analytic assessment of the literature. The selection and interpretation of studies therefore remain subject to potential selection bias and should be understood as part of an exploratory conceptual synthesis.

A further limitation concerns the heterogeneity of the evidentiary levels integrated in this review. Evolutionary hypotheses, neuroimaging findings, cognitive theories, molecular genetics, epidemiology, and intergenerational epigenetic studies do not constitute a single causal chain. Rather, they provide complementary but indirect perspectives on autism-related neurocognitive variability. Consequently, the proposed framework should not be interpreted as demonstrating that Asperger-associated traits were adaptively selected or that they currently represent adaptive traits in all individuals or contexts.

In addition, the phenotype considered in this review is not a discrete biological entity. The term “Asperger-associated traits” is used in a historical and descriptive sense, and the cited literature includes heterogeneous populations, including diagnosed ASD samples, individuals without intellectual disability, autistic adults, subclinical autistic traits, and theoretical cognitive profiles. This limits the generalizability of the conclusions and reinforces the need for future studies using more precise phenotypic stratification.

Another limitation concerns impairment and support needs. A strengths-based interpretation may be useful for counterbalancing purely deficit-oriented models, but it must not obscure the substantial functional difficulties, sensory distress, psychiatric comorbidities, communication challenges, and social exclusion experienced by many autistic individuals. The present framework should therefore be interpreted as compatible with clinical impairment, not as a replacement for clinical assessment or support-oriented models.

Finally, the discussion of intergenerational epigenetics remains preliminary. Current evidence is largely associative and mostly concerns ASD-related risk or quantitative autistic traits rather than specific Asperger-associated cognitive profiles. No causal inference can therefore be drawn regarding paternal exposures, germline epigenetic regulation, and the emergence of specific cognitive traits such as systemizing, persistence, technical imagination, or pattern-focused reasoning. For these reasons, the present review should be read as a conceptual synthesis intended to generate testable hypotheses, not as evidence that Asperger-associated traits represent an adaptive phenotype.

## Conclusions

In conclusion, this narrative review proposes a hypothesis-generating framework in which traits historically associated with Asperger syndrome are understood as context-dependent expressions of human neurocognitive variability rather than as a discrete diagnostic or biologically fixed profile. The reviewed literature supports a cautious interpretation in which traits such as systemizing, persistence, attention to detail, pattern-focused reasoning, technical interests, and lower reliance on implicit social cues may have different functional meanings depending on environmental demands, developmental trajectories, individual heterogeneity, and available supports.

However, the current evidence does not demonstrate that these traits were directly selected for, nor that they are universally adaptive. The models discussed in this review should be interpreted as theoretical, correlational, or preliminary contributions rather than as components of a single established causal pathway. Similarly, emerging intergenerational epigenetic findings remain associative and not specific to Asperger-associated profiles. Future research should test these hypotheses using longitudinal, genetically informed, neurobiologically grounded, and clinically well-characterized designs capable of distinguishing cognitive strengths, vulnerabilities, environmental fit, and functional outcomes across different autistic profiles.
